# Supplementary material for: Causal Relationship between Aging and Anorexia Nervosa: A White-Matter-Microstructure-Mediated Mendelian Randomization Analysis
Source: Biomedicines. 2024 Aug 16;12(8):1874. doi: 10.3390/biomedicines12081874 (PMC11351342; doi:10.3390/biomedicines12081874)
Supplement: Supplementary file 1 [file biomedicines-12-01874-s001.zip › Supplementary Figure/Figure S9/Leaveoneout plot of MR analysis for ubm-b-1975 to AN.pdf]

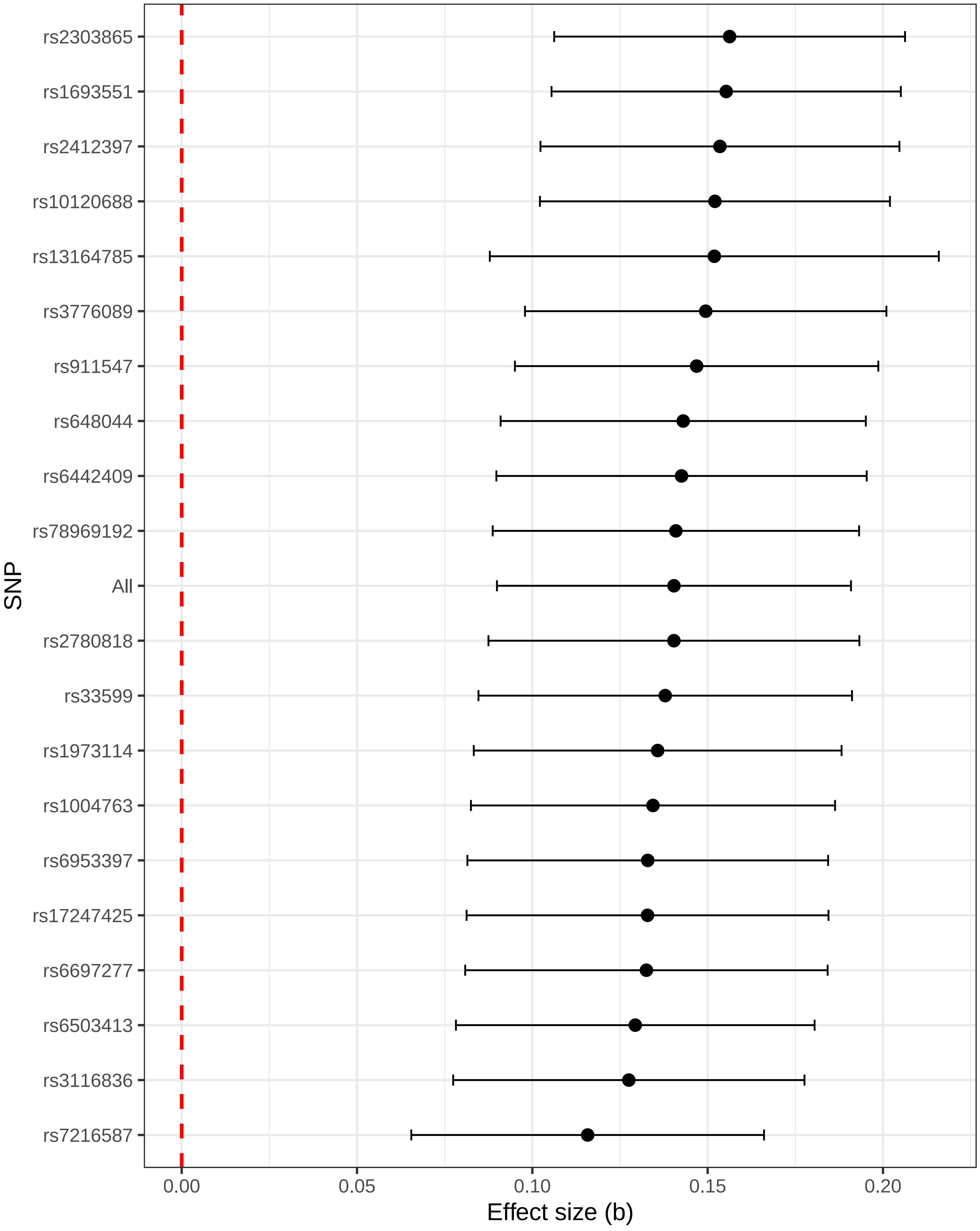
rs2303865
rs1693551
rs2412397
rs10120688
rs13164785
rs3776089
rs911547
rs648044
rs6442409
rs78969192
All
rs2780818
rs33599
rs1973114
rs1004763
rs6953397
rs17247425
rs6697277
rs6503413
rs3116836
rs7216587
SNP
0.00
0.05
0.10
0.15
0.20
Effect size (b)
